# Improved High-Frequency Ultrasound Corneal Biometric Accuracy by Micrometer-Resolution Acoustic-Property Maps of the Cornea

**DOI:** 10.1167/tvst.7.2.21

**Published:** 2018-04-11

**Authors:** Daniel Rohrbach, Ronald H. Silverman, Dan Chun, Harriet O. Lloyd, Raksha Urs, Jonathan Mamou

**Affiliations:** 1 Lizzi Center for Biomedical Engineering, Riverside Research, New York, NY, USA; 2 Department of Ophthalmology, Columbia University Medical Center, New York, NY, USA

**Keywords:** scanning acoustic microscopy, cornea, speed of sound, epithelium, stroma

## Abstract

**Purpose:**

Mapping of epithelial thickness (ET) is useful for detection of keratoconus, a disease characterized by corneal thinning and bulging in which epithelial thinning occurs over the apex. In prior clinical studies, optical coherence tomography (OCT) measurements of ET were systematically thinner than those obtained by 40-MHz high-frequency ultrasound (HFU) where a constant speed of sound (*c*) of 1636 m/s was used for all corneal layers. The purpose of this work was to study the acoustic properties, that is, *c*, acoustic impedance (*Z*), and attenuation (*α*) of the corneal epithelium and stroma independently using a scanning acoustic microscope (SAM) to investigate the discrepancy between OCT and HFU estimates of ET.

**Methods:**

Twelve unfixed pig corneas were snap-frozen and 6-μm sections were scanned using a custom-built SAM with an F-1.08, 500-MHz transducer and a 264-MHz bandwidth. Two-dimensional maps of *c*, *Z*, and *α* with a spatial resolution of 4 μm were derived.

**Results:**

SAM showed that the value of *c* in the epithelium (i.e., 1548 ± 18 m/s) is substantially lower than the value of *c* in the stroma (i.e., 1686 ± 33 m/s).

**Conclusion:**

SAM results demonstrated that the assumption of a constant value of *c* for all corneal layers is incorrect and explains the prior discrepancy between OCT and HFU ET determinations.

**Translational Relevance:**

The findings of this study have important implications for HFU-based ET measurements and will improve future keratoconus diagnosis by providing more-accurate ET estimates.

## Introduction

The human cornea is responsible for approximately two-thirds of the eye's optical refractive power, with the remainder provided by the crystalline lens.^1^ Clear vision is thus highly dependent on the optical quality of the cornea. Anatomically, the cornea is a layered structure with an approximately spherical anterior surface with a 7.8-mm radius at the central region of its anterior surface. The outermost layer is the epithelium (Ep), which is approximately 50 μm thick.^2^ Bowman's layer (BL) is a 10-μm-thick acellular basement layer at the interface of the Ep and stroma (St). St normally is approximately 500 μm in thickness and is composed largely of lamellae or belts of collagen that constitute 90% of the corneal thickness. The St is internally limited by Descemet's membrane (Dc) and a monolayer of endothelial cells that maintains corneal dehydration and clarity by active transport.

Accurate determination of the thickness of the cornea and its constituent layers is crucial for monitoring and detecting pathologies such as keratoconus.^3^ Keratoconus is a corneal disease affecting approximately 1 in 2000 people.^4^ It is characterized by progressive central corneal thinning and protrusion, and with progression to end-stage, may require corneal transplantation to preserve vision.^4^

If the corneal epithelium is removed, as in procedures such as riboflavin cross-linking and photorefractive keratectomy (PRK), it will regrow entirely to cover the cornea in about 1 week. In keratoconus, where the St progressively bulges forward, the epithelium will compensate by thinning over the stromal apex. As individuals with undiagnosed keratoconus often seek corneal refractive surgery to correct their worsening vision, the surgeon must carefully identify such cases because keratoconic corneas are already structurally compromised and removal of additional St will accelerate the disease process. Early identification of keratoconus can also be beneficial therapeutically, as UV-activated riboflavin collagen cross-linking can arrest progression.^5^

Current procedures for keratoconus screening rely on optical depiction of anterior surface topography typically obtained by computer analysis of concentric Placido rings projected onto the corneal surface and two-surface topography^6^ using scanning Scheimpflug imaging.^7^

Because of the epithelium's habit of masking irregularities in the underlying St, epithelial thickness (ET) mapping can help screen for early changes in the ET pattern resulting from stromal bulging in subclinical keratoconus before topographic changes become evident. Hence, mapping ET using optical coherence tomography (OCT)^8^ or high-frequency ultrasound (HFU)^9^ has become an additional method for keratoconus detection.

**Figure 1:**
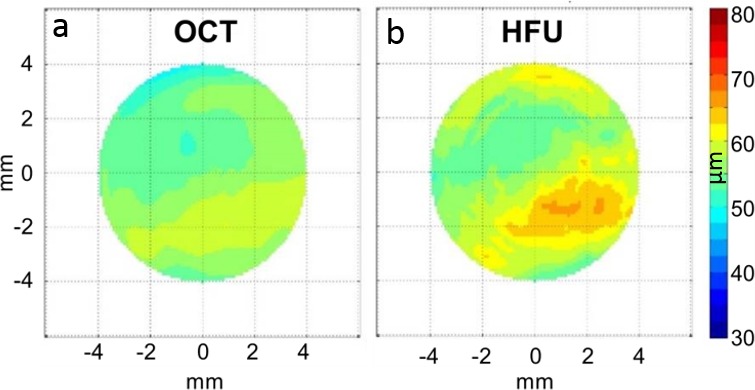
Comparative OCT (a) and HFU (b) ET maps of the right eye of a normal 64-year-old male subject. Note that although both OCT and HFU demonstrate a band of thickened epithelium inferiorly (but within normal limits) using the same color scale for both maps, the thickening is more evident in HFU than OCT.

In two independent studies comparing HFU and OCT,^10^ we assessed ET of more than 200 normal and post-laser-assisted in situ keratomileusis (LASIK) corneas.^11^ A significant difference between HFU and OCT-based thickness estimates was observed, which was largest in regions where the epithelium was thickest. Figure 1 shows an example of comparative HFU- and OCT-based ET maps of a normal cornea.

While a difference in measured ET between HFU and OCT was expected because the tear film is included in the OCT measurement, this would imply a thicker ET when measured by OCT. Surprisingly, the opposite was observed in both studies, with mean differences between HFU- and OCT-determined ET in the central cornea of normal eyes of +0.7 and +1.7 μm reported by Urs et al.^11^ and Reinstein et al.,^10^ respectively. Reinstein^10^ reported OCT-based ET to be 2.5 μm thinner on average than HFU when evaluating the thickened epithelium in 175 post-LASIK eyes (i.e., a thickness of 57.9 μm in post-LASIK versus 53.4 μm in untreated corneas).

Estimation of the thickness of each corneal layer by means of HFU depends on assumptions about their speed of sound. At 40 MHz, the Ep and St are readily resolved. However, to date their particular speeds of sound are unknown, and a fixed value of 1636 m/s has been used for the cornea as a whole and applied uniformly across all layers.

To assess the acoustical properties of the corneal structures, we utilized a custom-made scanning acoustic microscope (SAM). SAM, with frequencies between 100 MHz and 1.5 GHz providing resolutions between 30 and 1 μm, is a mature and reliable tool for assessing acoustical properties, for example, acoustic impedance and speed of sound, which are related to stiffness and mass density. SAM has been applied successfully to characterize various soft tissues such as skin, coronary artery, lymph node, prostate, tendon, liver, and muscle tissue.^12–20^ However, very few studies have reported SAM measurements for ocular tissues.^21–23^ Using SAM, Beshtawi et al.^21^ found a 5% increase of speed of sound of cross-linked corneal tissue compared to cryosectioned corneas and found good agreement between histology and speed of sound in the treated cases.^21^ Marmor et al.^22^ found significant variations of acoustic properties of retinal layers. Our group demonstrated that tissue mechanical properties at 7-μm resolution significantly differ among layers of murine retinas, indicating that structural changes are manifested in the mechanical properties of tissue at the micrometer level.^23^

In a preliminary study using a 250-MHz transducer and 7-μm lateral resolution, we scanned the corneas of two flash-frozen pig eyes to study the acoustical properties at the micrometer level.^24^ We found a significant lower speed of sound in Ep (i.e., 1539 ± 18 m/s) when compared to St (1591 ± 28 m/s). If these values are used to estimate ET instead of a global corneal speed of sound of 1636 m/s, then the difference in OCT and HFU measurements could be explained.

The aim of this study was to confirm these preliminary results on a larger data set using a novel 500-MHz, 4-μm resolution SAM that was recently developed by our group and tested on fixed, paraffin-embedded human ocular tissues.^25^ We decided to use our 500-MHz system on unfixed cryosectioned corneas because it would allow us to resolve and characterize corneal structures such as BL in humans, which is about 10–15 μm thick.

## Material and Methods

### Animal Model and Sample Preparation

Twelve pig eyes were shipped on ice overnight. The anterior segment was excised and mounted on a Barron Anterior Chamber (Katena Products, Inc., Denville, NJ), which was filled with normal saline and pressurized to ∼15 mm Hg. A Barron radial vacuum 9-mm diameter trephine was then used to excise the central cornea, which was incubated in 20% dextran overnight. The corneal button was then embedded in a compound (Tissue-Tek O.C.T.; Scigen Scientific, Gardena, CA) and flash-frozen. A cryostat (CM1950; Leica Biosystems, Nussloch, Germany) was used to produce 6-μm sections from the central cornea. The sections were transferred to a glass microscope slide and stored at −80°C. To thaw and rehydrate the sections before scanning, slides were placed successively in two normal saline baths for 5 minutes at room temperature. After SAM imaging, the sections were counterstained using hematoxylin and eosin and imaged by light microscopy. Studies were conducted in compliance with the ARVO Statement for Use of Animals in Ophthalmic and Vision Research.

### Acoustic Microscopy

Details about the working principle and parameter estimations of the SAM can be found in our previous publications.^23,25^ Briefly, the SAM was equipped with a custom-made F-1.08, 500-MHz transducer (Fraunhofer IBMT, Sulzbach, Germany) with a 264-MHz bandwidth and 4-μm lateral focal beam. The device uses a 500-MHz monocycle pulser (GEOZONDAS, Vilnius, Lithuania) to excite the ultrasound transducer, and radio-frequency signals were amplified using a 1-GHz bandwidth, 30-dB amplifier (MITEQ, Hauppauge, NY) and digitized at 2.5 GHz using a 12-bit oscilloscope (Teledyne LeCroy, Chestnut Ridge, NY). The specimens were scanned by mounting the microscope slide in an upside-down configuration on a three-axis, precision-scanning stage (Newport Corp, Irvine, CA). A drop of degassed saline was used as coupling fluid. Maps of sample thickness (*d*), speed of sound (*c*), acoustic impedance (*Z*), attenuation (*α*), bulk modulus (*K*), and mass density (*ρ*) were derived from the frequency domain representation of each recorded signal using a model-based approach as extensively described by Rohrbach, et al.^25^ Rare, unreliable estimates caused by scan artifacts were removed according to methods described in our previous studies.^23,25^

To compare our results with the findings from our preliminary study,^24^ we scanned three cornea sections using our 250-MHz SAM system after the sections were scanned with the 500-MHz SAM system. The F-1.16, 250-MHz transducer (Fraunhofer IBMT) has a 160-MHz bandwidth and 7-μm lateral resolution. A 300-MHz monocyle pulser (GZ1120ME-03; GEOZONDAS) was used to excite the transducer. Scanning stages and digitation were the same as for the 500-MHz SAM system.

### SAM Data Processing

Corneal tissues were identified using the coregistered histology and SAM amplitude map images. The amplitude images were generated from the maximum of the envelope signals. In our previous studies, the amplitude image showed good contrast comparable to the histology-stained images and thus is used to identify different tissues (see fig. 8 in Rohrbach et al.^25^ for comparison of histology and SAM images). In the present study, three layers were identified: Ep, St, and Dc. BL was not visible because in pigs the BL is less developed than in primates.

**Figure 2:**
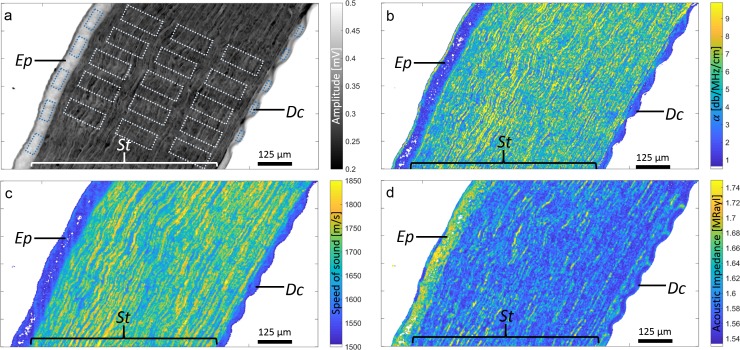
Representative acoustic-property maps at 500 MHz of unfixed pig cornea showing amplitude (a), attenuation (b), speed of sound (c), and acoustic impedance (d), showing tissues of Ep, St, and Dc.

St was further divided into anterior, middle, and posterior stroma (St_a_, St_b_, and St_c_). For each corneal layer (i.e., Ep, St_a_, St_b_, St_c_, and Dc), five rectangular regions of interest (ROI) were defined and equally distributed. Areas that showed preparation artifacts such as tissue folding or holes were avoided. Figure 2a shows an example of defined ROIs. For each ROI, mean and standard deviation for all parameters (i.e., *c*, *Z*, *α*, *K*, and *ρ*) were calculated.

N-way ANOVA followed by post hoc multicomparison Tukey-Kramer tests were used to evaluate the impact of corneal tissue and sample specific effects on the derived acoustical properties. A Kolmogorov-Smirnov test was used to test if parameter distributions are normally distributed. All statistical results were considered significant for *P* values less than 0.05. Relationships between the acoustical properties were assessed using linear regression analysis with a significance level of 0.05. All data points of the two-dimensional parameter maps were included in the regression analysis. The statistical computations were conducted using the MATLAB Statistics Toolbox (MathWorks, Natick, MA).

### In Vivo HFU Ultrasound and OCT Scans

**Figure 3:**
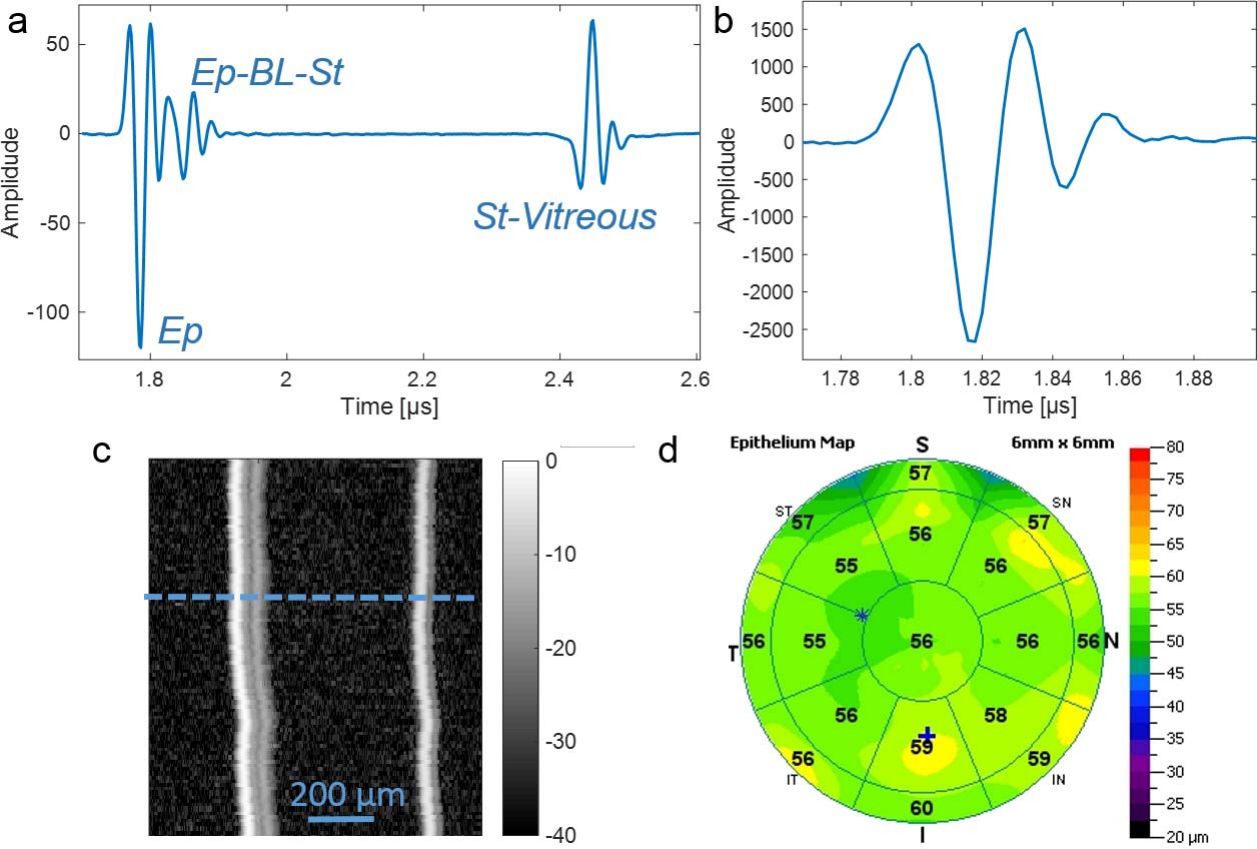
(a) Representing specular reflection from the anterior echo complex of a normal human cornea, signal shows reflection from Ep, Ep-BL-St interface, and St-vitreous interface. (b) Glass plate echo. (c) B-mode image of HFU scan. Dotted line indicates measured signal in (a). (d) OCT-based ET map of the same human subject.

In the previous clinical studies, HFU data were acquired using Artemis 2^10^ (Arcscan, Inc, Golden, CO). The device uses a broadband single-element polymer transducer with a center frequency of 38 MHz and 12.8-mm focal length. Scans are performed mechanically moving the transducer in an arc such that the corneal surface is in the focal plane at normal incidence to the ultrasound beam axis during the scan. To compare the acoustic properties that were derived ex vivo using our SAM to acoustic properties in in vivo conditions, in this study we estimated the acoustic impedances and speed of sound of the Ep from HFU data from three normal human subjects. Three standard cornea-protocol scans (256 vectors over a 70° arc with 2048, 8-bit axial samples at 500-MHz sampling frequency) were performed in the horizontal meridian for each eye. This provided an approximate scan depth of 3.2 mm and width of 11 mm in the focal plane. Scans were centered on the corneal vertex based on simultaneous camera views of the pupil and adjustment for maximum echo amplitude, occurring in the focal plane at normal incidence of the ultrasound beam axis on the corneal surface. Scans were analyzed to find the ET, *c*, and *Z* using the same approach as for our acoustic microscope.^23,25^ Briefly, the reflected signals comprising reflections from the Ep surface and Ep-BL were modeled and fit by a summation of two reference signals with different phase and amplitude. Reference signals were obtained from measurements of a glass marble with an acoustic impedance of 14.05 mega Rayl (MRayl). By mapping the reflected signals into the frequency domain using a fast-Fourier-transform (FFT) algorithm and by normalizing with the FFT of the reference signal, sample thickness, speed of sound, and *Z*, can be calculated directly.^26^ Examples of one of the HFU scans and the reference glass plate signal are depicted in Figure 3a–c.

Furthermore, OCT measurements were performed using an anterior segment OCT (RTVue; Optovue, Inc., Fremont, CA) to compare estimates of ET in one eye of the three normal human subjects. The measured ETs were used to estimate the speed of sound in Ep from the HIFU measurements. An example scan is shown in Figure 3d.

The HFU and OCT experiments adhered to the tenets of the Declaration of Helsinki, and informed consent was obtained from the subjects after explanation of the nature and possible consequences of the study. The research was approved by the institutional review board of Columbia University Medical Center, New York.

## Results

### Artemis Measurements

The in vivo value of epithelial acoustic impedance (*Z_E_*) for the three subjects was found to be 1.64, 1.62, and 1.65 MRayl, which leads to an average in vivo *Z_E_* value of 1.64 MRayl. Using the central OCT ET measurements (i.e., 56, 53, and 54 μm for the three subjects), the speed of sound in the Ep was found to be 1631, 1565, and 1579 m/s, which leads to an average speed of sound of 1592 m/s. The thickness was corrected for the tear film, which was assumed to be 2 μm thick.

### Scanning Acoustic Microscopy

**Figure 4:**
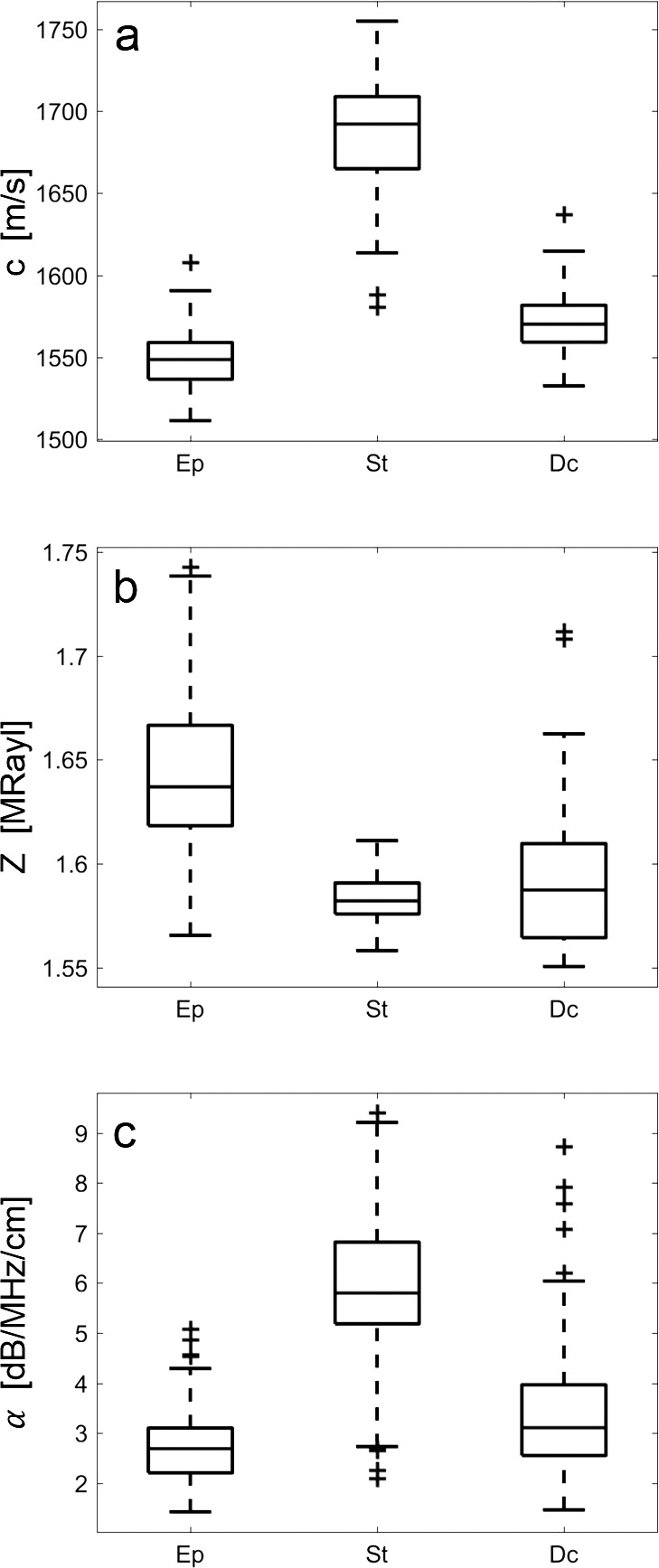
Box plots showing acoustic-property variations, that is, speed of sound (a), acoustic impedance (b), and attenuation (c) of Ep, St, and Dc tissue. Dotted lines indicate significant differences.

Representative maps of signal amplitude and acoustic properties (i.e., *c*, *Z*, and *α*) for a single pig cornea are depicted in Figure 2. The resolution afforded by the 500-MHz SAM system was sufficient to distinguish the corneal Ep, St, and Dc. The acoustic-property values show a strong contrast between Ep and St with lower *c* and larger *Z* values in Ep. Average values of each tissue for all acoustic properties are summarized in Table 1. ANOVA revealed significant variation of all parameters among the three tissue layers (i.e., Ep, St, Dc). The Ep showed lowest c and α, but highest *Z* values. Figure 4 shows box plots of *c*, *Z*, and *α* among Ep, St, Dc. No significant impact of St region (i.e., anterior to posterior) was found for any of the acoustical parameters (Table 2).

**Table 1:**
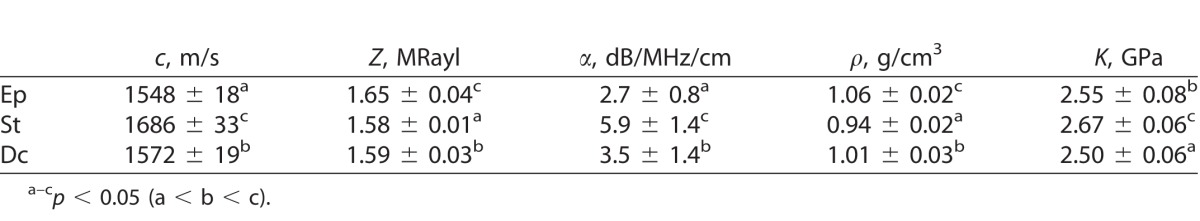
Average Acoustic Properties of Ep, St, and Dc

**Table 2:**
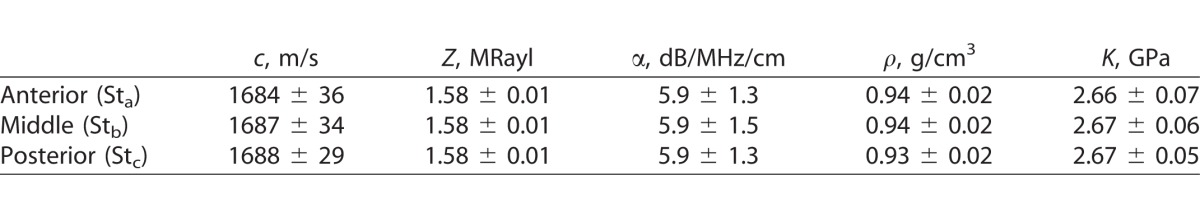
Average Acoustic Properties of Anterior, Middle, and Posterior Regions of St, With No Significant Differences Among the Regions Found

**Figure 5:**
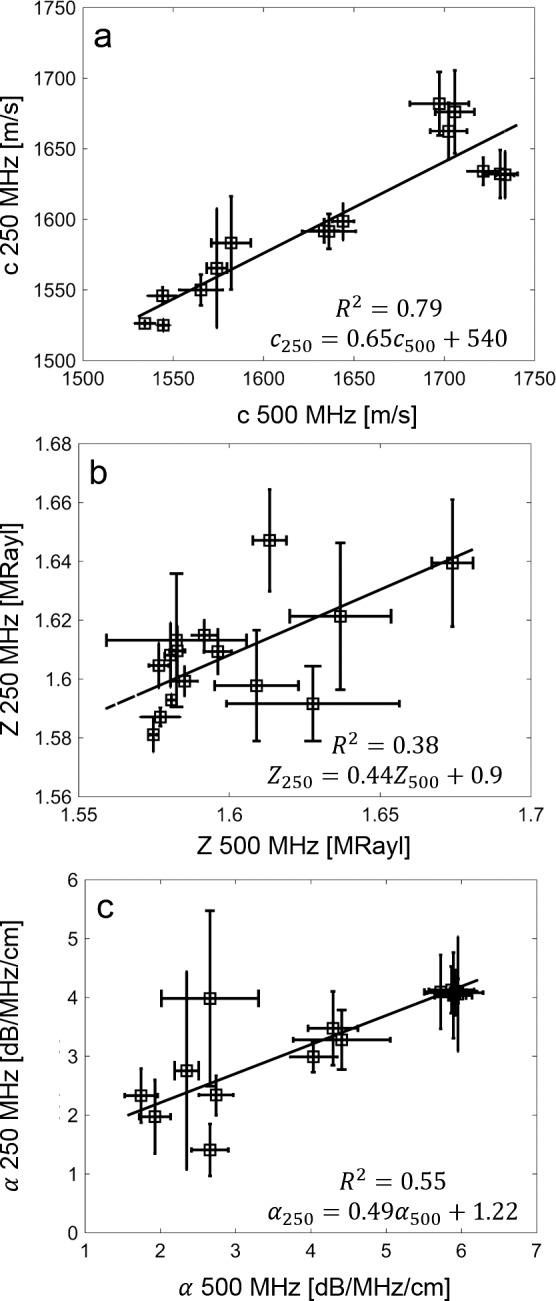
Linear correlation between 500- and 250-MHz measurements of speed of sound (a), acoustic impedance (b), and attenuation (c).

On average, the 250-MHz data showed significantly lower values than the 500-MHz data for all acoustical parameters (i.e., Δ*c* = −37 m/s, Δ*Z* = −0.01 MRayl, Δα = −0.87 dB/MHz/cm, ΔK = −0.04 GPa) except for *ρ*, which showed significantly higher values (Δ*ρ* = +0.03 m/s). Note *Z* had the smallest *P* value (i.e., 0.045) and was smaller than the standard deviation of the mean (SD) values of the 250-MHz (i.e., SD = 0.02 MRayl) and 500-MHz (i.e., SD = 0.03 MRayl) values. To further test the relation of the acoustical parameters when compared between the 250- and 500-MHz transducers, we conducted linear regression analysis. Significant positive correlation (i.e., when predicting 250- from 500-MHz data) was found among all acoustical parameters. However, only *c*, *K*, and *ρ* showed strong correlation (*R*^2^*_c_* = 0.79, *R*^2^*_K_* = 0.79, *R*^2^*_ρ_* = 0.78, *P* ≤ 0.01). The other parameters showed moderate correlation (*R*^2^_α_ = 0.55, *R*^2^*_Z_* = 0.34, *P* ≤ 0.01). Figure 5 shows the correlation plots for *c*, *Z*, and *α*. Table 3 provides correlation coefficients among the acoustical parameters. The strongest correlations were found between *c* versus *K*, *Z* versus *K*, and *Z* versus *ρ*. Correlations between *c* versus *Z* and *K* versus *ρ* were moderate. Correlations with *α* and sample thickness were weak. The correlation of *c* versus *ρ* was weak as well.

**Table 3:**
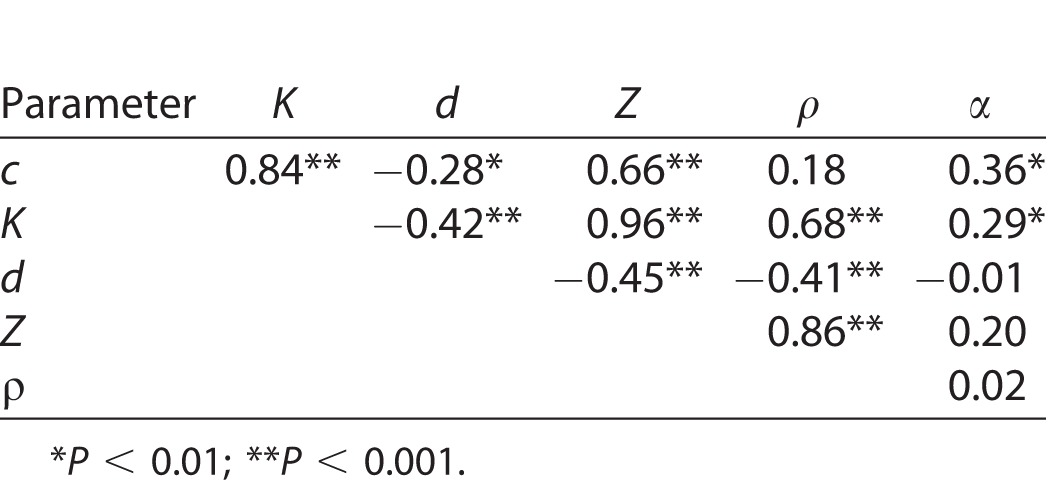
Correlation Coefficient (R) of Linear Correlation Between Acoustical Parameter

## Discussion

To the best of our knowledge, this is the first quantitative and comprehensive study reporting quantitative assessment of acoustical properties of different corneal layers with a very fine resolution. Only one other study, to our knowledge, used an acoustic microscopy approach to assess the speed of sound in the cornea. Beshtawi et al.^21^ reported a speed of sound of 1584 m/s of cryosectioned human corneas measured with a 761-MHz transducer. Another study used lower frequencies (i.e., 20-MHz center frequency)^27^ and found average speed of sound values of human and pig corneas^27^ of 1572 m/s and 1588 m/s, respectively. Both studies are in good agreement with the average values (i.e., 1602 ± 23 m/s) found in this study. Furthermore, if the speed of sound values of this study (see \newcommand{\bialpha}{\boldsymbol{\alpha}}\newcommand{\bibeta}{\boldsymbol{\beta}}\newcommand{\bigamma}{\boldsymbol{\gamma}}\newcommand{\bidelta}{\boldsymbol{\delta}}\newcommand{\bivarepsilon}{\boldsymbol{\varepsilon}}\newcommand{\bizeta}{\boldsymbol{\zeta}}\newcommand{\bieta}{\boldsymbol{\eta}}\newcommand{\bitheta}{\boldsymbol{\theta}}\newcommand{\biiota}{\boldsymbol{\iota}}\newcommand{\bikappa}{\boldsymbol{\kappa}}\newcommand{\bilambda}{\boldsymbol{\lambda}}\newcommand{\bimu}{\boldsymbol{\mu}}\newcommand{\binu}{\boldsymbol{\nu}}\newcommand{\bixi}{\boldsymbol{\xi}}\newcommand{\biomicron}{\boldsymbol{\micron}}\newcommand{\bipi}{\boldsymbol{\pi}}\newcommand{\birho}{\boldsymbol{\rho}}\newcommand{\bisigma}{\boldsymbol{\sigma}}\newcommand{\bitau}{\boldsymbol{\tau}}\newcommand{\biupsilon}{\boldsymbol{\upsilon}}\newcommand{\biphi}{\boldsymbol{\phi}}\newcommand{\bichi}{\boldsymbol{\chi}}\newcommand{\bipsi}{\boldsymbol{\psi}}\newcommand{\biomega}{\boldsymbol{\omega}}\(c\) in Table 1) are considered together with assumed corneal tissue thicknesses of ET ∼50 μm, St thickness ∼500 μm, and Dc thickness ∼12 μm,^2^ then the compound speed of sound of the entire cornea would yield 1670 ± 31 m/s, which is close to the commonly used speed of sound value of 1636 m/s and is well in the range of the speed of sound values reported in previous studies.^3,27,28^ However, none of these studies reported the speed of sound in St and epithelium separately. The results of the present study confirm our preliminary results^24^ and indicate that the speed of sound in Ep is significantly lower when compared with speed of sound in the St. This has important implications for current ultrasound-based layered corneal biometry where ET has heretofore been based on the assumption that speed of sound in all corneal layers is the same.

However, our results indicate that the speed of sound of the Ep is lower than that of St. In our previous preliminary study using a 250-MHz transducer, we found an average Ep speed of sound of 1539 ± 18 m/s.^24^ When considering the high correlation of speed of sound values between 500 and 250-MHz measurements (i.e., *R*^2^ = 0.79) and applying the linear relationship to the values found in this study, the corrected speed of sound value of 1542 ± 18 m/s agrees with our previous findings. Because of dispersion effects occurring in soft tissues, speed of sound values can vary slightly with ultrasound frequency. Typically, in soft tissues, speed of sound values increase with frequency. The increase can be exactly quantified using the derivative of the frequency-dependent attenuation.^29^ In future studies, we will accurately estimate the speed of sound values for the in vivo applications at 40 MHz based on those measured at 250 and 500 MHz using the frequency dependence of the attenuation, which can also be measured using SAM.

If the SAM-based *c*_Ep_ of 1542 ± 18 m/s is multiplied by a factor of 1.0216 to account for speed of sound at body temperature versus room temperature (assuming the temperature dependency of water from 23°C to 37°C), then the data indicate *c*_Ep_ ≈ 1575 m/s in vivo. Until now, nothing was known regarding *c*_Ep_, and the current accepted value for *c* in the cornea (i.e., 1636 m/s) is relatively high compared to typical *c* values in soft tissues. We would expect a lower value of *c*_Ep_ compared to *c*_St_ since the Ep is a cellular tissue in contrast to the St, which is mostly collagenous. In a previous clinical study,^10^ we found that the difference between OCT- and HFU-based ET scaled with ET. In fact the data suggest that a lower value of *c*_Ep_ would improve the correspondence between layer thicknesses that are estimated by OCT and HFU. Regression analysis between OCT- and HFU-based measurements of ET resulted in a best-fit value of 1573 m/s for *c*_Ep_, which is very close to the value found in this study. This is further supported by the in vivo HFU findings of this study with an average *c*_Ep_ of 1592 m/s.

These findings suggest that ET values previously obtained by ultrasound systematically overestimated ET by ∼4%, or approximately 2 μm for a 50-μm epithelium. Both studies found the central ET in normal eyes to be 1–2 μm greater when determined using ultrasound compared to OCT, which initially seemed inexplicable because OCT also includes the tear film thickness in its estimation of ET. However, with the above findings, this discrepancy is explained, that is, if a \newcommand{\bialpha}{\boldsymbol{\alpha}}\newcommand{\bibeta}{\boldsymbol{\beta}}\newcommand{\bigamma}{\boldsymbol{\gamma}}\newcommand{\bidelta}{\boldsymbol{\delta}}\newcommand{\bivarepsilon}{\boldsymbol{\varepsilon}}\newcommand{\bizeta}{\boldsymbol{\zeta}}\newcommand{\bieta}{\boldsymbol{\eta}}\newcommand{\bitheta}{\boldsymbol{\theta}}\newcommand{\biiota}{\boldsymbol{\iota}}\newcommand{\bikappa}{\boldsymbol{\kappa}}\newcommand{\bilambda}{\boldsymbol{\lambda}}\newcommand{\bimu}{\boldsymbol{\mu}}\newcommand{\binu}{\boldsymbol{\nu}}\newcommand{\bixi}{\boldsymbol{\xi}}\newcommand{\biomicron}{\boldsymbol{\micron}}\newcommand{\bipi}{\boldsymbol{\pi}}\newcommand{\birho}{\boldsymbol{\rho}}\newcommand{\bisigma}{\boldsymbol{\sigma}}\newcommand{\bitau}{\boldsymbol{\tau}}\newcommand{\biupsilon}{\boldsymbol{\upsilon}}\newcommand{\biphi}{\boldsymbol{\phi}}\newcommand{\bichi}{\boldsymbol{\chi}}\newcommand{\bipsi}{\boldsymbol{\psi}}\newcommand{\biomega}{\boldsymbol{\omega}}\({c_{EP}}\sim \)1572 m/s is assumed instead of the commonly used global value of 1636 m/s, then the thickness estimates from 40-MHz HFU and OCT matches. In the future, appropriate corrections can be applied retrospectively to existing as well as to new data. The systematic nature of this effect means that it has no impact on findings regarding ET patterns associated with keratoconus. Rather, it accounts well for differences in values estimated in previous HFU and OCT ET studies.

The values of *Z*_Ep_ found in this study are notably higher than those found in our previous preliminary study at 250 MHz.^24^ The correlation was weak (i.e., *R*^2^_Z_ = 0.34) between 250- and 500-MHz estimates of *Z*. However, if the linear relationship that was found in this study is used to correct the values of the Ep, the impedance would become *Z*_E_ = 1.63 ± 0.02 MRayl at 250 MHz, which is still larger than that found in our preliminary study (i.e., 1.58 ± 0.03 MRayl). Nevertheless, the values obtained in this study are well in accordance with estimated in vivo values (i.e., 1.64 MRayl).

**Figure 6:**
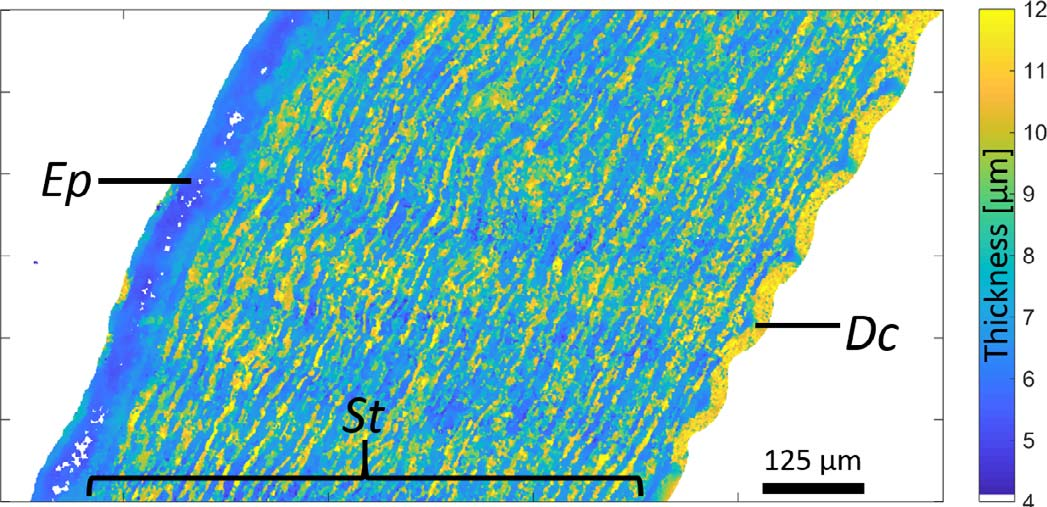
Map of sample thickness.

We found *ρ* values < 1 g/cm^3^ in the St. These values are likely to be erroneous because St mainly consists of type I collagen, and the density of the porcine cornea has been reported to be 1.062 ± 0.005 g/cm^3^.^30^ The low *ρ* values can be explained by the variation in *d*_St_ of the cornea samples. Figure 6 shows a map of the sample thickness (*d*) of the same sample as depicted in Figure 2. Consistently throughout all specimens, we observed the same significant thickness-variation pattern (between 6 and 12 μm) even though the cryotome was set to 6 μm. In an ideal situation, the cut of the thin, 6-μm-thick sections would result in a smooth surface. However, as observed in Figure 2, this assumption does not hold for corneal St. We hypothesize that the variation in thickness can be explained by the major orientation of collagenous lamellae. In pigs, the St is composed of lamellae with thicknesses of about 0.2–2 μm^31,32^ and interlaced in way that is similar to a plywood structure.^33,34^ The lamellae have different preferred fibril orientations and might be affected differently during the cutting procedure. If this is the case, such a structure would lead to surface scattering and nonperpendicular reflections of the ultrasound waves leading to smaller recorded amplitudes and thus to smaller estimates of acoustic impedance. In fact, our results indicate a strong dependency of *Z* and *ρ*. Since *ρ = Z/c*, incorrect lower *Z* estimates would directly lead to lower estimates of *ρ*. Therefore, the results of this study, which are related to the stromal tissue, need to be used with caution. Further studies should aim to either improve sample preparation procedures to produce smooth sample surfaces or to assess the sample surface roughness using complementary modalities such as atomic force microscopy.^35^ This information could be used in conjunction with numerical ultrasound simulations to develop a novel acoustic parameter estimation algorithm, properly taking into account surface scattering effects.

Furthermore, the results obtained using the 250-MHz SAM system need to be considered with caution. A *d* of 6-μm is close to the axial resolution of the system and may cause more erroneous results. Currently, we are investigating different signal-processing algorithms that will improve parameter estimation close to the axial resolution of the SAM system.

Nevertheless, as shown in Figure 6, the surface of the Ep, measured using the 500-MHz SAM system, is comparatively smooth and has the expected section thickness of approximately 6 μm. Hence, the results found for the Ep are not affecte d by the thickness-variation–related effects seen in the St. Furthermore, we did not experience any significant variation of the acoustic parameters as a function of scanning time that would have resulted from tissue swelling, temperature variations, or evaporation of the coupling medium. Because our SAM scans lines in the *x* direction successively, a time-dependent effect would have resulted in a gradient of the acoustical parameters along the *y* direction. However, such an effect never occurred, as illustrated in Figure 2. From our previous SAM studies on ocular tissues,^23–25,36^ we were able to maintain an approximately constant temperature with variations of less than 1°C even for scanning times as long as 40 minutes. The average scan area of the samples in this study was less than 4 mm^2^ and required scanning times of less than 20 minutes. Therefore, we expect the coupling-medium temperature to have no effect on the acoustic-property-value estimate in this study. In fact, the measured acoustical-property values of the epithelium agree with values estimated from in vivo data.

These results indicate that using SAM to measure speed of sound values of Ep resolved the discrepancy between HFU-based epithelium thickness estimates compared to OCT-based estimates (i.e., larger thickness values occur in HFU-based estimates if a global \newcommand{\bialpha}{\boldsymbol{\alpha}}\newcommand{\bibeta}{\boldsymbol{\beta}}\newcommand{\bigamma}{\boldsymbol{\gamma}}\newcommand{\bidelta}{\boldsymbol{\delta}}\newcommand{\bivarepsilon}{\boldsymbol{\varepsilon}}\newcommand{\bizeta}{\boldsymbol{\zeta}}\newcommand{\bieta}{\boldsymbol{\eta}}\newcommand{\bitheta}{\boldsymbol{\theta}}\newcommand{\biiota}{\boldsymbol{\iota}}\newcommand{\bikappa}{\boldsymbol{\kappa}}\newcommand{\bilambda}{\boldsymbol{\lambda}}\newcommand{\bimu}{\boldsymbol{\mu}}\newcommand{\binu}{\boldsymbol{\nu}}\newcommand{\bixi}{\boldsymbol{\xi}}\newcommand{\biomicron}{\boldsymbol{\micron}}\newcommand{\bipi}{\boldsymbol{\pi}}\newcommand{\birho}{\boldsymbol{\rho}}\newcommand{\bisigma}{\boldsymbol{\sigma}}\newcommand{\bitau}{\boldsymbol{\tau}}\newcommand{\biupsilon}{\boldsymbol{\upsilon}}\newcommand{\biphi}{\boldsymbol{\phi}}\newcommand{\bichi}{\boldsymbol{\chi}}\newcommand{\bipsi}{\boldsymbol{\psi}}\newcommand{\biomega}{\boldsymbol{\omega}}\({c_{EP}}\) of 1636 m/s is assumed; see Fig. 1) and demonstrate the importance of accurately accounting for acoustic-tissue-property differences when making HFU-based corneal-thickness measurements.
